# MicroRNA Mediated Cardioprotection – Is There a Path to Clinical Translation?

**DOI:** 10.3389/fbioe.2020.00149

**Published:** 2020-03-20

**Authors:** Timo Z. Nazari-Shafti, Vasileios Exarchos, Héctor Rodriguez Cetina Biefer, Nikola Cesarovic, Heike Meyborg, Volkmar Falk, Maximilian Y. Emmert

**Affiliations:** ^1^Department for Cardiovascular and Thoracic Surgery, German Heart Center Berlin, Berlin, Germany; ^2^Berlin Institute of Health, Berlin, Germany; ^3^Deutsches Zentrum für Herz-und Kreislauferkrankungen, Berlin, Germany; ^4^Department of Health Sciences and Technology, ETH Zürich, Zurich, Switzerland; ^5^Clinic for Cardiovascular Surgery, Charité Universitätsmedizin Berlin, Berlin, Germany; ^6^Institute for Regenerative Medicine, University of Zurich, Zurich, Switzerland; ^7^Wyss Zurich, University of Zurich and ETH Zurich, Zurich, Switzerland

**Keywords:** microRNA, extracellular vesicles, second generation cell therapies, translation, cardioprotection, secretome

## Abstract

In the past 20 years, there have been several approaches to achieve cardioprotection or cardiac regeneration using a vast variety of cell therapies and remote ischemic pre-conditioning (RIPC). To date, substantial proof that either cell therapy or RIPC has the potential for clinically relevant cardiac repair or regeneration of cardiac tissue is still pending. Preclinical trials indicate that the secretome of cells *in situ* (during RIPC) as well as of transplanted cells may exhibit cardioprotective properties in the acute setting of cardiac injury. The secretome generally consists of cell-specific cytokines and extracellular vesicles (EVs) containing microRNAs (miRNAs). It is currently hypothesized that a subset of known miRNAs play a crucial part in the facilitation of cardioprotective effects. miRNAs are small non-coding RNA molecules that inhibit post-transcriptional translation of messenger RNAs (mRNAs) and play an important role in gene translation regulation. It is also known that one miRNAs usually targets multiple mRNAs. This makes predictability of pharmacokinetics and mechanism of action very difficult and could in part explain the inferior performance of various progenitor cells in clinical studies. Identification of miRNAs involved in cardioprotection and remodeling, the composition of miRNA profiles, and the exact mechanism of action are important to the design of future cell-based but also cell-free cardioprotective therapeutics. This review will give a description of miRNA with cardioprotective properties and a current overview on known mechanism of action and potential missing links. Additionally, we will give an outlook on the potential for clinical translation of miRNAs in the setting of myocardial infarction and heart failure.

## Introduction

Cardiovascular disease remains one of the most important challenges clinicians face today. Despite huge efforts in prevention and undeniable progress in acute and short-term survival, disease progression over long-term still burdens our health care system without a real curative approach. The reason: regenerative capacity of an adult human heart is quite limited due to the low turnover rate of cardiomyocytes and lack of a sufficient pool of tissue resident progenitors (Tzahor and Poss, 2017). This renders the human heart very susceptible to any form of acute or chronic injury with a critical loss of cardiomyocytes or their function, ultimately leading to the clinical manifestation of heart failure. For these facts, cardiac medicine has been identified as a promising field for application of regeneration technologies. In the past 20 years, cell-based therapies have aimed to either induce ‘*de novo*’ generation or stimulate tissue dormant progenitors to differentiate into mature cardiomyocytes (Behfar et al., 2014). The promising results from *in vitro* studies and pre-clinical trials have led to a large number of clinical trials that for the most part investigated the therapeutic effect of different bone marrow, adipose- or neonatal tissue derived progenitor cells (first generation cell therapy). With limited pre-clinical data available in hindsight, a rush into first clinical trials in the early and mid 2000s has largely failed to demonstrate any meaningful results for patients with cardiac disease. More recently, myocardium resident cardiac progenitor cells, iPSCs, and conditioned progenitor cells have also been investigated in that context (second generation cell therapy) (Cambria et al., 2017). However, they as well failed to reach respective primary endpoints in most clinical trials; that is to say reduction in scar size and stabilization or improvement of cardiac function (Moyé, 2014; Gyöngyösi et al., 2015). As a result, the idea of a regenerating heart has been abandoned by many scientists. Luckily, the failure of such clinical studies has also led to *post hoc* analyses of the mechanism of action by which various cell types exhibit cardioprotection in the injured heart. Today, there is consensus that paracrine mediators, released by transplanted cells upon injury signals, mediate protection and limit adverse myocardial remodeling (Madonna et al., 2019). There is evidence emerging, that mediators can limit the extend of cardiomyocyte loss during acute injury and positively impact adverse myocardial remodeling in the chronic setting. Within the past 10 years, microRNAs (miRNAs) have come into focus as the next generation “cell” therapy studies have demonstrated that the paracrine secretion of nano- and macrovesicles containing miRNAs are mainly responsible for the cardioprotective effect of cellular therapies. Preclinical trials were able to demonstrate that miRNAs or extracellular vesicles (EVs) containing miRNAs were capable to reproduce the cellular effects of cardioprotection (Behfar and Terzic, 2019; Madonna et al., 2019; Maring et al., 2019). Within the scope of this review, we intend to provide a general overview of the potential role of miRNAs in cardioprotection and elaborate on potential use of miRNAs as a therapeutic agent.

## Micro RNAs – One Shoe Fits All?

**FIGURE 1:**
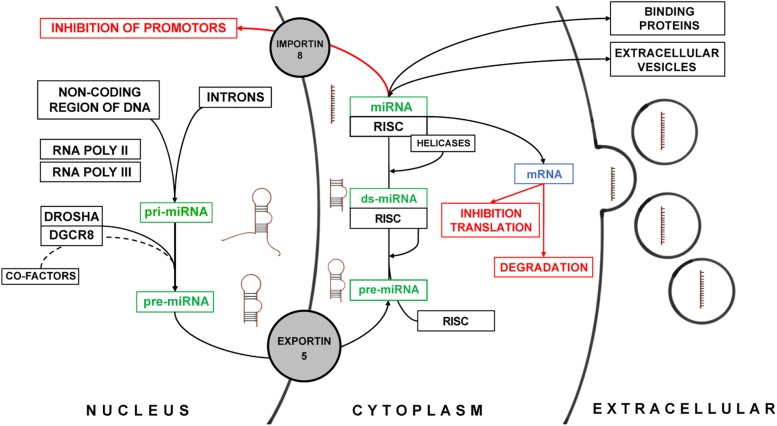
Summary of the biosynthesis and the biological effect of miRNAs. The different stages of miRNA are schematically depicted next to the green description of miRNA. The biological effect of miRNAs is marked in red.

MicroRNAs play an important role in the inhibition of messenger RNA (mRNA) translation in the cytoplasm but have been also identified to regulate transcription in the nuclear compartment of mammalian cells (Bartel, 2004). The biosynthesis was long believed to be linear and universal for all miRNAs. However, recent functional studies demonstrated, that a multitude of alternate miRNA-specific biosynthesis pathways exist and that they require a plethora of regulatory mechanisms – many of which still need to be identified (Lee et al., 1993; Bartel, 2004) (Figure 1). miRNA are encoded within the entire genome and are usually arranged in clusters (Rodriguez et al., 2004). Most miRNA genes are located in the non-coding areas of the genome. In some cases, miRNAs are located within the introns of protein coding genes such as miR-103 which is located within in the intron of pantothenate kinase 1, 2, and 3 together with miR-107 (Rodriguez et al., 2004; Lin et al., 2006). miRNAs are transcribed by RNA Polymerases II and III into a primary miRNA (pri-miRNA). The pri-miRNA consists of a terminal loop region, a stem and two single-stranded flanking RNA regions up- and downstream of the hairpin (Cai et al., 2004; Lee et al., 2004). For the canonical pathway of miRNA maturation, the terminal single stranded RNA region is then spliced by the complex of Drosha and DGCR8 protein (DiGeorge critical region 8) (Lee et al., 2003). The DGCR8 protein has a binding and proof-reading domain that ensures the correct binding and identification of the cleavage site for the Drosha protein, a RNase III enzyme. For some miRNAs additional factors may be required for the correct splicing of the hairpin precursor (Guil and Cáceres, 2007; Davis et al., 2008; Michlewski et al., 2008) before the precursor miRNA (pre-miRNA) is transported from the nucleus into the cytoplasm by Exportin-5 (Yi et al., 2003). Exportin-5 not only acts as a transporter but also as an additional “proof-reader” only transporting correctly processed pre-miRNA (Zeng and Cullen, 2004). In the cytoplasm, the pre-miRNA – still consisting of the terminal loop and the double stranded stem – binds to the RNA-induced silencing complex (RISC) (Gregory et al., 2005). This protein complex contains RNase Dicer, double stranded RNA binding domain proteins (Tar RNA binding protein), PACT (protein activator of PKR), and Argonaute-2 (MacRae et al., 2008). The latter is the key component that mediates the miRNA inhibition of transcription of mRNAs (Diederichs and Haber, 2007). The terminal loop of pre-miRNAs is spliced off, whereas for some of the miRNAs both the guide and passenger strand can serve as individual mature miRNAs (Matranga et al., 2005). In general, the passenger strand is degraded after unwinding of the double stranded pre-miRNA by helicases (Leuschner et al., 2006). Similar to the processing of miRNAs in the nucleus some miRNAs are dependent on additional co-factors for maturation (Hutvágner et al., 2001; Diederichs et al., 2008). This highlights again, that miRNA biosynthesis is subject to an integrate regulatory machinery which we only just begin to understand. Upon maturation, miRNAs as part of the RISC complex either inhibit the translation of their target mRNA or they are packaged into EVs contained in multivesicular bodies to be released in the extracellular space (Turchinovich et al., 2012). The encapsulation of miRNAs does not happen at random and is also a highly regulated process (Turchinovich et al., 2012; Villarroya-Beltri et al., 2013). This allows for the dynamic response of cells in regard to which miRNAs are released upon microenvironmental cues. A study by Villarroya-Beltri et al. (2013) has shown that specific motifs within the sequence of miRNA determine their localization in either the cytoplasm or EVs. They have also identified heterogeneous nuclear ribonucleoproteins such as hnRNPA2B1 and hnRNPA1 that specifically bind to these motifs and are also present in EVs. Independent of their origin, all miRNAs have in common to alter gene expression via inhibitory mechanisms of posttranscriptional modification of mRNAs. The mechanism of inhibition depends on the complementarity of the miRNA sequence to its target mRNA. miRNAs with high complementarity direct the RISC to the mRNA and initiate the degradation. Lower complementarity can lead to inhibition of ribosomal translation of the target mRNA. It is overall hypothesized that the specificity of miRNAs is determined by the quality and stability of base pairing to their respective targets. Furthermore, miRNAs have also been identified to perform transcriptional silencing by targeting promotor regions within the heterochromatin. The aforementioned regulation of biosynthesis, maturation, and biological effects of numerous miRNA can be influenced by changes in the cellular microenvironment in a non-linear manner – posing a major challenge for scientists to predict possible effects of miRNA *in vivo* (Lee et al., 1993; Liu B. et al., 2014).

## Cardioprotective Mirnas – a Two-Sided Sword

For some time now, it has been hypothesized that factors released by cells under stress can induce a protective effect in neighboring or remote tissues (Kharbanda et al., 2002; Chen et al., 2008; Hausenloy et al., 2015). We now know that the key player in transmitting these signals are miRNAs. In the extracellular compartment, miRNAs are usually transported via EVs or binding proteins to protect them from degradation by nucleases (Li et al., 2012; Boon and Dimmeler, 2015). Similarly, the effect of therapeutic cell preparations exhibits their protective effect via the transmission of EVs loaded with miRNAs. Over the course of the past 10 years the sera of patients undergoing an ischemic event as well as the secretory profile of most cells utilized for cardioprotective purposes have been characterized (Baglio et al., 2015; Barile et al., 2016; Barile and Vassalli, 2017; Shao et al., 2017; Bellin et al., 2019). Each year, an increasing number of pathways are identified that either hint toward protection or damage of the myocardium (Barile et al., 2014, 2016; Gallet et al., 2016; Ciullo et al., 2019). Many functional studies have preceded these in-depth analyses of miRNA as cardioprotective agents, remote ischemic pre-conditioning (RIPC) being one of the most prominent examples (Kharbanda et al., 2002; Hausenloy et al., 2015). Here, a different organ or the heart itself is exposed to brief, non-fatal ischemia/reperfusion. Pre-clinical models show that RIPC can increase the survival of cardiomyocytes upon injury and positively impacts the myocardial remodeling (Konstantinov et al., 2005; Wei et al., 2011). In RIPC, circulating miRNA and EVs play an important role (Frey et al., 2018; Spannbauer et al., 2019). Even though circulating miRNAs and EVs have been identified as key mediators of that cardioprotective effect, it is a rather crude and a non-targeted therapeutic approach. Most when RIPC was investigated in clinical trials, none of the primary endpoints predicted by preclinical studies were met (Brevoord et al., 2012). Similar to experience from clinical trials investigating cell therapies, the exact mechanisms of action of RIPC were not fully understood and may explain the failed translation. However, the data collected on miRNAs from these studies laid the groundwork for many functional studies investigating the cardioprotective effects of miRNAs.

In recent years, a plethora of mechanistical studies for the downstream effect of various miRNA were conducted. Here, miRNAs were either investigated as diagnostic markers or as potential targets for therapies (Barile et al., 2016). The in-depth analysis of regulation of the identified miRNAs and their downstream targets revealed that some miRNAs had contradictory effects in the myocardium (Table 1). Furthermore, to the best of our knowledge no study performed any concurrent analysis to identify potential targets that are not related to a cardioprotective effect. In this next section, we highlight a selection of miRNAs that have been associated with a cardioprotective potential but also bear the risk of adverse or off-target effects. Most of these miRNAs were identified either in EVs of therapeutic cell products or as biomarkers during acute and chronic myocardial injury. The knowledge of their exact mechanism of action is therefore highly recommended. The following miRNAs are only a small example of miRNAs that are commonly identified but not limited to their cardioprotective potential. The selection of miRNA should not be seen as a comprehensive summary of all known cardioprotective miRNAs which would be beyond the scope of this review. Others have provided more detailed lists of cardioprotective miRNAs (Varga et al., 2015; Wendt et al., 2018). The pre-clinical experience with the following miRNAs should highlight the potential and pitfalls we as scientist may face when designing therapeutic strategies with miRNAs.

**TABLE 1:** Summarizes a selection of miRNAs that have been identified with either a harmful or cardioprotective property in cardiovascular disease.

miRNA	Disease model	Releasing cell type	Experimental approach	Experimental model	Effect	Identified targets	Recipient cell	Off-target effects	PMID
miR-665	I/R	CM	Suppression of miR-665 via dexmedetomidine	Rat heart Langendorff preparation	Improved LVDP during reperfusion	AK1, Cbr2	Cardiac cells	Not investigated	31026731
	HF	–	I.m. injection with anti-sense miRNA plasmids	Rat model of HF	Improved LVEF, reduced CM apoptosis, improved Mc ultrastructure	GLP1R	Cardiac cells	Not investigated	30666648
	HF	Global	I.v. injection of rAAV miR-665 inhb.	Murine model of LV pressure overload	Improved LVEF, reduced fibrosis, improved vascularization	CD34	Global	Not investigated	30243022
	–	Human CM	*In vitro* gain and loss of function in human CM	Mechanistic model	–	Cbr1 and Cbr2	Human CM	–	25111814
miR-132	I/R	–	Loss of function *in vivo*, gain of function *in vitro*	Murine hind limb ischemia	Slower perfusion recovery, less collateralization, modulation of RAS-MAPK signaling	Rasa1 and Spred1	–	Not investigated	25016614
	Afib	–	*In vitro* loss and gain of function in CF	Mechanistic model	In human and dog with Afib decreased expr. miR-132 in atrium	CTGF	CF	–	28731126
	DCM	–	*In vitro* analysis of cardiac cell isolates from DCM rats, overexpression of miR-132	DCM rat model	Activation of PI3K/Akt pathway, CM apoptosis down	PTEN	–	–	30271437
	AMI	BM-MSCex electroporated with miR-132	I.m. injections with MSCex	Murine model of AMI	Increased LVEF, enhanced neovascularization in BZ	Rasa1	HUVECS	Not investigated	30216493
miR-132 + miR-126	DMap	–	Transfection of aortic rings with miR-132, miR-126	Endothelial sprouting in aortic rings under high glucose	Decreased EC apoptosis, improved endothelial sprouting	Spred1	HUVECS, Ecs	–	31179325
miR-210 + miR-132 + miR-146a-3p	AMI	CPCs	I.v. injection with CPCex rich in miR-210, miR-132, miR-146a-3p vs. Fibex	Murine model of AMI	Less CM apoptosis, enhanced angiogenesis in BZ, improved LVEF	EFNA3, PTP1b	–	Not investigated	28731126
miR-126	AMI	AT-MSCs overexpressing miR-126	I.m. injection of AT-MSCex	Murine model of AMI	Increased neoangiogenesis	Not investigated	–	Not investigated	29241208
miR-126-5p	Endothelial injury	–	KO of EC Dicer and rescue experiment with miR-126-5p transfection	CA injury	Endothelial Dicer processes pre-mir-126 into mir-126-3p (guide strand) and the passenger strand-5p. 5p is involved in dendothelial repair and proliferation by targeting the Notch1 inhibitor Delta-like homolog 1 (Dlk1)	Dlk1	ECs	Not investigated	30213595
mir-210	AMI	–	Observational study	AMI in rats	Increased levels of miR-210	–	–	–	31596148
	AMI	BM-MSCs	BM-MSCs rich in miR-210 vs. BM-MSCs with miR-210 silencing	Murine model of myocardial infarction	Increase LVEF, increased neoangiogenesis	EFNA3	–	Not investigated	28249798
	I/R	BM-EPCs	BM-EPCs gain and loss of miR-210	Murine hind limb ischemia	With miR-210 improved perfusion recovery and collateralization	EFNA3	ECs	Not investigated	29908843
	I/R	–	Loss and gain of function	*In vitro* in H9c2 cells, mechanistic model		CXCR4	H9c2	–	29710553
mir-206	Afib		Lentiviral overexpression of miR-206 in PVFP	Canine model of Afib	Overexpression of miR-206 increased incidence of Afib	GCH1	–	Not investigated	29436714
	Afib	–	Overexpression of miR-206 in murine hearts	Transgenic mouse model	Overexpression led to decreased lifespan and arrhythmias	Cx43	–	Not investigated	30322759
	AMI	–	Cardiac specific expression of miR-206	Murine model of AMI	CM hypertrophy and increases survival under AMI	FBPP1	–	Not investigated	26333362
	HF	–	Increased expression of miR-206 via HMGB1	Murine model of AMI	Increased collagenolytic activity, decreases myocardial fibrosis	TIMP3	CF	Not investigated	21731608
	AMI	–	*In vitro* loss and gain of function, with *in vivo* confirmation	Murine model of AMI	Reduced CM apoptosis, improved LVEF	ATG3	H9c2	Not investigated	30551524
miR-206 + miR-216b	AMI	–	Via HDC gain and loss influence expression of miR-206, miR-216b	Murine model of AMI	Targets Atg13 and reduces autophagy upon hypoxia. miR-206 is induced via histamine	ATG3	–	Not investigated	29880830
miR-206 + miR-1	DMap	–	*In vitro* loss and gain of function	*In vitro* in H9c2 cells, mechanistic model	Increased CM apoptosis	Hsp60	H9c2	–	20655308
miR-146a	DCM	CF, CM	AAV9 mediated overexpression of miR146a *in vivo* and *in vitro*	Murine model of LV pressure overload	Decreased myocardial contractility	SUMO1	CM	Not investigated	30355233
	AMI	EPCs	EPC injection in BZ	AMI in rats	Downregulation of miR-146a and reduced CM apoptosis and increased VEGF expression	–	–	Not investigated	30344699
	–	–	Lentiviral overexpression of miR-146a in H92c	*In vitro* in H9c2 cells, mechanistic model	Increases MMP9, may reduce fibrosis in injured heart	FOS, AP1	CM	–	26112171
	Sepsis induced cardiac dysfunction	–	Transfection of mice with miR-146a, *in vitro* H9c2 and macrophages	Murine sepsis model	Attenuation of sepsis induced myocardial dysfunction	IRAK, TRAF6	H9c2, J774 macrophages		26048146
	DoxDCM	CM	*In vitro* overexpression and suppression of miR-146a	*In vitro* in H9c2 cells, mechanistic model	Induction of cell death upon Dox treatment	ErbB4	H9c2	–	20495188
	AMI	AT-MSCs overexpressing miR-146a	I.m. injections of AT-MSCex native and overexpressing miR-146a	AMI in rats	Decreased CM apoptosis, decreased inflammation, decreased fibrosis	EGR1	H9c2	Not investigated	30362610
miR-146a-5p	DoxDCM	CPCs	CPCex rich in miR-146a-5p vs. Fibex	DoxDCM model in rats	Decreased CM apoptosis	Traf6, Smad4, Nox4, Mpo	–	Not investigated	31098627
miR-146a + miR-155	–	DC	Injection of endotoxin exposed mice with Dcex rich in miR-155 and miR-146a	Murine model of endotoxin inflammation	miR-146a attenuates inflammation, miR-155 increases inflammation	–	–	Not investigated	26084661
miR-22	AMI	–	AAV9 overexpression of miR-22	AMI in rats	Decreased CM apoptosis, decreases infarct size	CBP	–	Not investigated	24338162
	–	–	Overexpression of miR-22 in murine lungs, zebrafish and ECs *in vitro*	–		VEC	EC	Not investigated	28112401
	AMI, HF	–	Gain and loss study on miR-22	*In vitro* in CF, mechanistic model	Overexpression limits expression of Col1α1, Col3α1	TGFβR	CF	–	27997889
	AMI	–	Gain and loss study on miR-22	*In vitro* in rat CMs	Overexpression prevented autophagy and apoptosis in CMs	p38α	CM	–	
	AMI	–	I.m. injection of miR-22 inhib., loss and gain function *in vitro*	AMI in rats	Inhibition decreases infarct size, reduces CM apoptosis	Sirt1, PGC1α	H9c2	Not investigated	27174562
	DCM	–	miR-22 deficient mice and gain and loss function in H9c2	Murine model of left ventricular pressure over load	miR-22 suppression led to left ventricular dilation	PReBPb	H9c2	Not investigated	22570371
	DCM	–	Gain and loss of miR-22 in mice	Murine model of left ventricular pressure overload	Overexpression of miR-22 protected from DCM	Sirt1, Hdac4	–	Not investigated	23524588
	–	–	Gain and loss study in H9c2	*In vitro* in H9c2 cells, mechanistic model	Prevents the activation of NFkB/Caspase3 mediated apoptosis upon stress	p65	H9c2	–	30504734
	AMI	–	*In vitro* gain and loss study, *in vivo* miR-22 KO mice	Murine model of AMI	In miR-21 KO mice decreased survival, decreased LVEF, increased scar size	KBTBD7	Macrophages		29991775
miR-21	AMI	–	AAV9 overexpression of miR-21	AMI in rats	Promotes cardiac fibroblast activation and CF to myofibroblast transformation (CMT)	Jagged1	–	Not investigated	29808534
	–				Targets PDCD4 to reduce apoptosis after HIF-1alpha activated expression of mir21				29170412
	AMI	–	*In vitro* in CF, *in vivo* induction of miR-21 via TGF-β1	Murine model of AMI	Increased fibrosis in the heart upon AMI	Smad7	CF	Not investigated	28817807
	–	–	Gain and loss study in H9c2 cells	*In vitro* in H9c2 cells, mechanistic model	Inhibits autophagy and apoptosis upon I/R partially via the Akt/mTOR pathway	–	H9c2	–	27680680
	–	–	Exposure of H9c2 cells with CPC derived EVs rich in miR-21	*In vitro* in H9c2 cells, mechanistic model	Targets PDCD4 when CDC derived exosomes are added to CMs	–	H9c2	–	27336721
	–	–	Gain and loss study in PBMCs	*In vitro* in human PBMCs, mechanistic model	Via targeting SMAD7, mir-21 can reduce the number of circulating Tregs	Smad7	Human Tregs	–	26383248
	–	–	Gain and loss study in H9c2 cells	*In vitro* in H9c2 cells, mechanistic model	Proof for a positive feedback loop between mir-21 and HIF-1alpha which reduces apoptosis upon hypoxia and stress	PTEN	H9c2	–	24983504
miR-21-5p	–	–	Gain and loss study in H9c2	*In vitro* in H9c2 cells, mechanistic model	Modulation of reliance on glycolytic or fatty acid oxidation in mitochondria	–	H9c2	–	30657727
	–	BM-MSCs	Exposure of H9c2 cells with BM-MSCex rich in miR-21a-5p	*In vitro* in H9c2 cells, mechanistic model	Reduction of CM apoptosis upon stress. This was identified in EVs from MSCs (miR-21a-5p).	PDCD4, PTEN, Peli1 an dFasL	H9c2	–	29698635

*AAV, adeno-associated-virus; Afib, atrial fibrillation; AMI, acute myocardial infarction; AT-MSCex, AT-MSC exosomes; AT-MSCs, adipose tissue derived mesenchymal stem cells; BM-EPCs, bone marrow derived endothelial progenitor cells; BM-MSCex, BM-MSC exosomes; BM-MSCs, bone marrow derived mesenchymal stem cells; CF, cardiac fibroblast; CPCs, cardiac progenitor cells; DCex, dendritic cell exosomes; DCM, dilated cardiomyopathy; DCs, dendritic cells; DMap, diabetic myocardial microangiopathy; DoxDCM, doxorubicin induced dilated cardiomyopathy; ECs, endothelial cells; EPCs, endothelial progenitor cells; HF, heart failure; HUVECs, human umbilical vein endothelial cells; I/R, ischemia reperfusion.*

### MicroRNAs – The Good, the Bad, and the Ugly

#### Off-Target Effects of miRNAs

Especially studies investigating RIPC have identified clusters of cardioprotective miRNAs (Varga et al., 2015). In these studies as well as in those that used EVs, an undefined cocktail of miRNAs was systemically applied. In both cases, defining the mode of action is virtually impossible. Usually, hundreds of miRNAs can be identified in RIPC and even in EV preparations numerous miRNAs can be found, some of which taken by themselves have been identified as damaging to the myocardium. As an example, miR-665 has been identified in sera and serum exosomes of patients with heart failure (Li et al., 2016; Fan et al., 2019). MiR-665 directly targets the cannabinoid receptor 2 (CbR2) and adenylate kinase 1 (AK1) (Möhnle et al., 2014; Lin et al., 2019). In a rat heart Langendorff preparation, inhibition of CbR2 and AK1 leads to the upregulation of pro-apoptotic genes such as B cell lymphoma 2 (Bcl-2) and Bcl-2-associated X protein (Bax) and caspase-3 in H9c2 cells, a commonly used rat cardiomyocyte cell line (Yu et al., 2019). In a murine model of myocardial infarction, AAV9 transfection with miR-665 antisense miRNA led to improvement of cardiac function (Fan et al., 2018). Here, the group has identified glucagon-like peptide-1 receptor (GLP1R) as the target for miR-665. Inhibition of miR-665 expression led to increased cAMP signaling in the heart via the promotor GLP1R and reduced apoptotic events upon ischemia/reperfusion injury in the heart. Identification of harmful miRNAs like miRNA-665 in autologous exosome preparations from patients with heart failure could serve as quality markers and help prevent potential off-target effects. In some cases, off target effects or cardioprotective miRNAs have already been identified. miRNA-206, for example, has been associated with both cardioprotective, but also damaging effects upon overexpression (Table 1). Transcription of miR-206 can be induced via histamine release upon myocardial stress in mice (Ding et al., 2018). *In vitro*, miR-206 can prevent apoptosis in hypoxic conditions by targeting autophagy related protein 2 (ATG3) (Kong et al., 2019). In a murine model of acute myocardial infarction, this prevented ubiquitination of cytosomal proteins and apoptosis (Ding et al., 2018). By cardiac-specific overexpression of miR-206 is has been shown that forkhead box protein P1 induces hypertrophy of cardiomyocytes which during stress can prevent cardiomyocyte apoptosis (Yang et al., 2015). By inhibiting metalloproteinase inhibitor 3 (TIMP3), miR-206 has also been shown to attenuate cardiac fibrosis in the setting of chronic heart failure (Limana et al., 2011). However, two independent groups have also uncovered two mechanisms by which miR-206 increases the risk of cardiac arrhythmias. miR-206 targets connexin 43 (Cx43), an important component of gap junctions in the myocardium in a transgenic mouse model (Roell et al., 2007). Low expression of Cx43 has been associated with cardiac arrhythmias such as atrial fibrillation. Additionally, Wei et al. (2018) uncovered that miR-206 binds to GTP cyclohydrolase I (GCH1) in a canine model of atrial fibrillation (Afib). GCH1 is the rate limiting enzyme in *de novo* synthesis of tetrahydrobiopterin (BH4). Decreased expression of BH4 was associated with shortened refractory times in atrial cardiomyocytes in humans, which led to Afib (Wei et al., 2018). In this example, different groups were able to demonstrate a cardioprotective effect of miR-206. At the same time potential off target effects were uncovered that could impede the translation of important signal transduction proteins in the myocardium. These off-target effects were never investigated in the murine models that investigated the cardioprotective effects for this miRNA. It has also been shown that miRNA have species specific effects. A recently published large animal study demonstrated that the delivery of miR-199 via adenoviral transfer to the myocardium of pigs resulted in improved contractility and myocardial mass in the short term (Gabisonia et al., 2019). After 1 month, however, the pigs died of arrhythmias. Histological analysis revealed that the myocardium was infiltrated with proliferating cells displaying a poorly differentiated myoblastic phenotype. This off-target effect of miR-199 was not identified in small animal studies. Results like these raise the question whether results from murine models or even porcine models are really translatable into clinical applications without additional safeguards that can predict these off-target and adverse effects.

#### Neoangiogenesis – Good for Cardioprotection, Bad for Cancer Progression

Neoangiogenesis plays an important role in protecting the myocardium in the border zones from infarcts from myocardial remodeling. Therefore, many miRNAs such as miR-132, miR-126, and miR-210 have been investigated for their angiogenic potential (Table 1). The transcription of most of these miRNAs is dependent on the expression VEGF (Lei et al., 2015; Chodari et al., 2019). For instance, miR-132 expression is dependent in the promotor cAMP response element-binding protein as the transcription factor, which is induced by VEGF stimulation (Chodari et al., 2019). EVs from cardiac progenitor cells (CPCs) rich in miR-132 inhibit the translation of Ras GTPase activating protein (p120RasGAP) which promotes proliferation and sprouting of endothelial cells thus improving neovascularization *in vivo* and *in vitro* (Barile et al., 2014). In a murine model of acute myocardial infarction this led to improved left ventricular ejection fraction on a functional level and improved vascularization in the infarct border zone on a histological level (Barile et al., 2014). Similarly, EVs from adipose-derived mesenchymal stem cells (AT-MSCs) that overexpressed miR-126, also improved cardiac function and resulted in a denser microvasculature in the infarct border zone in rats (Luo et al., 2017). Functional studies have shown that miR-126 is highly depended on the activation of endothelial dicer RNA Polymerase III. Only the passenger strand miR-126-5p can bind to the Notch1 inhibitor Delta-like homolog 1 (Dlk1) (Zhou Z. et al., 2018). The notch signaling pathway is crucial to endothelial cell differentiation and endothelial sprouting (Mack and Iruela-Arispe, 2018). While there is no direct evidence linking the cardioprotective effect of miR-132 to cardiomyocytes, there is evidence that miR-132 can also reduce fibrosis in by targeting connective tissue growth factor (Zhang C.-J. et al., 2018). Furthermore, miR-132 has also been identified in a rat model of DCM to target phosphatase and tensin homolog (PTEN) (Ma et al., 2018). Suppression of PTEN activates the phosphoinositide 3-kinases/protein kinase B pathway (PI3K/Akt-pathway) which facilitates cardiomyocyte and endothelial proliferation alike (Zhang C.-J. et al., 2018). miR-210 has also been shown to promote angiogenesis in the myocardium (Mutharasan et al., 2011). In contrast to miR-126 and miR132, the expression of miR-210 depends on HIF-1alpha, which is also released under hypoxic stress (Barile et al., 2014). In both murine models of acute myocardial infarction and hind limb ischemia it has been shown that miR-210 encapsulated by EVs promotes angiogenesis in endothelial cells as well as suppresses apoptosis in cardiomyocytes by targeting Ephrin A3 (Barile et al., 2014; Wang et al., 2017; Besnier et al., 2018). While the upregulation or substitution of all aforementioned miRNAs are associated with a pro-angiogenic profile in the setting of myocardial infarction, they have also been identified in cancer biogenesis and metastasis formation. Also, in the field of cancer biology the data for these miRNAs is heterogeneous and in depending on cancer type they are both associated as a positive and negative prognostic marker. The heterogeneity of these results and their role in tumor progression could, however, pose an obstacle for their use as a cardioprotective agent. As a solution, patients susceptible to certain cancers that depend on overexpression of these miRNA need to be identified to prevent adverse effects from a hypothetical therapeutic miRNA.

#### How to Deal With Contradictory Results

miR-146a is part of a negative feedback loop in the canonical pathway of NFkB activation. miR-146a binds to the mRNA encoding for interleukin-1 receptor associated kinase 1 (IRAK1) and tumor necrosis factor receptor-associated receptor 6 (TRAF6). Both of this receptor bound factors are essential for the IL-1 and TNFalpha activation of NFkB (Fish and Cybulsky, 2015; Gao et al., 2015; Milano et al., 2019). As part of that negative feedback loop miR-146a is highly expressed in atherosclerotic plaques. However, the overexpression of this miRNA can also attenuate the inflammatory response as shown in gain and loss studies in ApoE deficient mice, where miRNA-146a plays an important part in the attenuation of atherosclerotic plaques (Fish and Cybulsky, 2015). In a rat model of AMI, EVs from AT-MSCs overexpressing miR-146a targeted the early growth response protein 1 in cardiomyocytes decreasing cardiomyocyte apoptosis, cardiac fibrosis and ultimately improving the heart function (Pan et al., 2019). Milano et al. (2019) demonstrated that the passenger strand miR-146a-5p reduced the inflammatory signaling pathways by directly targeting TRAF6, SMAD4, IRAK1, NADPH oxidase 4 (NOX4), and myeloperoxidase (MPO) in a model of doxorubicin/trastuzumab induced cardiomyopathy. Interestingly, another group showed that with treatment of doxorubicin alone an upregulation of miR146a occurs, targeting the receptor tyrosine-protein kinase erbB-4 (ErbB4). Here, a negative correlation between miR-146a and cardiac function was confirmed and explained by the suppression of the ErbB4 dependent neuregulin1/ErbB pathway, which is essential for adult cardiac function (Horie et al., 2010). It is, however, important to note that these results were only obtained from *in vitro* experiments. Both miR-21 and -22 have been associated with cardioprotective properties by reducing cardiomyocyte apoptosis and have been found in most EVs from therapeutic cell product isolates to date (Barile et al., 2016; Gallet et al., 2016). While there is evidence, that miR-22 also exhibits an anti-fibrotic effect during myocardial remodeling, overexpression of miR-21 is clearly associated with promoting cardiac fibrosis (Table 1). miR-21 directly targets jagged1 and SMAD7 in rat hearts when overexpressed via AAV9 (Zhou X.-L. et al., 2018). In the aforementioned experiment, jagged1 suppression activates cardiac fibroblast proliferation and facilitates cardiac fibroblast to myofibroblast transformation. In a murine model of AMI, suppression of SMAD7 via miR-21 led to an increased expression of Collagen 1 alpha, alpha-smooth muscle actin (alpha-SMA) and F-actin. In a similar model of AMI in rats, AVV9 mediated overexpression of miR-22 let to the inhibition of CBP-associated factor AP1 (Yang et al., 2014). Downregulation of this promotor leads to the activation of MMP9, which in turn can reduce cardiac fibrosis. Additionally, miR-22 binds to the mRNA coding for the TGFbeta receptor 1 (Hong et al., 2016). In mice, silencing of miR-22 led to increased expression of Collagen 1 alpha 1 and 3alpha1 and an overall increased amount of cardiac fibrosis after myocardial infarction. Regarding the anti-apoptotic effect of miR-22, there are contradictory results published to date. Gurha et al. (2012) and Du et al. (2016) both demonstrated that SIRT1 and PGC1alpha are both targeted by miR-22 in cardiomyocytes. In both experiments, overexpression of miR-22 lead to increased cell death and reduction in myocardial mass whereas in the studies published by Huang et al. (2013) and Hong et al. (2016) cardiac mass increased upon miR-22 overexpression and ischemia/reperfusion injury. Huang et al. (2013) also demonstrate the targeting of SIRT1 but in their hands, cardiomyocyte hypertrophy was the predominant finding. Hong et al. (2016) demonstrated decreased apoptosis levels in the myocardium and linked that effect to the inhibition of CREB. For miR-21 the anti-apoptotic effects is shown more robustly between different research teams (Table 1). miR-21 expression is under direct control of HIF1alpha but can also influence the expression of HIF1alpha itself in a positive feedback loop (Liu Y. et al., 2014). The miRNA targets programmed cell death protein 4 (PCDP4) and reduces cell apoptosis upon hypoxic stress. This effect has been demonstrated with EVs containing high levels of miR-21 from different cell types such as CPCs and MSCs (Xiao et al., 2016; Luther et al., 2018). Both miR-21 and miR-22 exemplify the various outcomes that can be seen when working with miRNAs in pre-clinical models. Even within one species there are variations and sometime contradictory results that can be elaborated by other groups on both ends of the aisle. Especially miRNAs that can promote fibrosis can have severe and unwanted effects in the injured heart and may lead to increase in scar mass. Here, patient screening and good patient selection may help to prevent these contradictory results. This can only be achieved by understanding all pathways that can be altered by each respective miRNA and tools to identify patients that may be susceptible to treatments with certain miRNAs.

## Summary

The data that has been collected on miRNAs targeting cardioprotection so far exemplifies the importance of knowing the relevant targets of miRNAs, since introduction of foreign miRNAs via exosomal transfer or other clinical relevant approaches may have some severe side effects. In this context, it is also worth noting that all of the aforementioned miRNAs are also involved in tumor biology. Especially miRNAs that impact neoangiogenesis in myocardial repair are important factors in tumor angiogenesis as well. In addition, most of the mechanistical studies have focused on the interaction of one miRNA on multiple targets. Upon ischemia/reperfusion or transfer of exogenous EVs the interaction of numerous miRNAs on multiple potential targets needs to be taken into account. With current methods, experimental data usually depicts a single linear arm in a complex matrix of interactions between miRNAs, mRNAs and transcription factors. Our current understanding of these complex matrices is only rudimental at best. And it may also be one of the main reasons why none of the clinical trials with cell-based therapies or RIPC have delivered the expected results we anticipated from pre-clinical experience. As repeatedly highlighted by numerous experts in the field, understanding the pathways by which a single- or a collection of miRNAs in an exosome, facilitate cardioprotection will be crucial for successful clinical translation (Madonna et al., 2016, 2019; Behfar and Terzic, 2019). Combined effort of computational models and artificial intelligence in merging and interpreting the acquired data, might help us in the future to achieve this goal. Identifying such ‘pathway’ matrices will be detrimental in defining quality standards for therapeutic exosome or single miRNA-based products. The past experience from cell-based therapies have taught us that preclinical data in the field of cardiac regeneration or cardioprotection does not necessarily translate into therapeutic success in the clinical setting. Going forward, we will have to deepen our understanding of miRNA interactions by strengthen our efforts to collaborate with bioinformaticians for more sophisticated predictive algorithms.

## Author Contributions

TN-S and ME contributed to the conception and design of this review. VE, HR, and HM helped to organize the article database and supported the literature review. TN-S wrote the first draft of the manuscript. All authors contributed to manuscript revision, read and approved the submitted version.

## Conflict of Interest

The authors declare that the research was conducted in the absence of any commercial or financial relationships that could be construed as a potential conflict of interest.
